# New Molecular Mechanisms and Markers in Inflammatory Disorders

**DOI:** 10.3390/ijms25126506

**Published:** 2024-06-13

**Authors:** Elena Vianello, Emanuela Galliera

**Affiliations:** 1 Department of Biomedical Sciences for Health, Università degli Studi di Milano, 20122 Milan, Italy; 2 Experimental Laboratory for Research on Organ Damage Biomarkers, IRCCS Istituto Auxologico Italiano, 20095 Cusano Milanino, Italy; 3 IRCCS Istituto Ortopedico Galeazzi, 20157 Milan, Italy

Inflammation is the primary response of different disorders, and these encompass a wide range of conditions in various tissues and organs [1]. Over the years, research has revealed numerous molecular mechanisms and markers associated with different inflammatory disorders [2]. Ongoing research in this field continues to uncover novel insights that may lead to improved treatments and better management of these complex and heterogeneous conditions. In this Special Issue, our aim was to collect current knowledge of the main hallmarks of inflammatory diseases, with the aim of understanding them and suggesting new therapeutic strategies and diagnostic tools to counteract the onset and progression of inflammatory states.

The term “biomarker” was first coined in 1973 by Rho et al. and was used to indicate the presence of material of biological origin in an article published in the *Space Life Sciences* journal [2]; however, thanks to Order et al., only in 1977 was the term associated for the first time with clinical use in a publication concerning tumor biomarkers, breast malignancy, and the clinical course of diseases, with this highlighting the importance and application of biomarkers in the medical field [3].

In 2000, the term “biomarker” was defined by the U.S. National Institutes of Health (NIH) as “a characteristic that is objectively measured and evaluated as an indication of normal and pathologic responses to a therapeutic intervention” [4]; recently, the term has been revised and redefined as “a functional variant or quantitative index of biological process that predicts or reflects the evolution of or predisposition to a disease or a response to therapy” [5]. In this regard, the field of biomarkers has expanded and has prompted research to discover new surrogate markers of clinical endpoints or pharmaceutical treatments thanks to their advantages in laboratory practice, as they require simpler and less expensive procedures for measurement than final clinical endpoints and, additionally, a shorter period of time for analysis.

**Figure 1:**
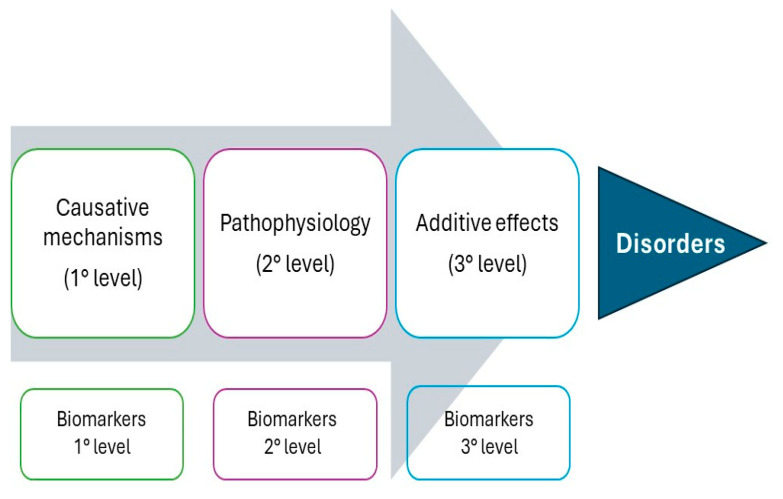
A representation of the time course of the mechanisms leading to a disease and their associated biomarkers in each evolution step. This image is a revisitation of the concept of the causative mechanisms of diseases proposed by Aroson et al. in 2008 [15].

For this reason, in the last decade, circulating biomarkers have come to play a crucial role in the diagnosis, prognosis, monitoring, and management of various diseases, including metabolic disorders, cancers, cardiovascular diseases, degenerative diseases, bone disorders, and infectious diseases [6]. Serum biomarkers are measurable substances that can indicate the presence or severity of a disease, provide insights into disease mechanisms, and guide treatment decisions for different kinds of pathologies, better characterizing the evolution of each disorder in so doing (Figure 1). Moreover, circulating biomarkers play a multifaceted role in modern medicine, providing valuable information across the continuum of care from diagnosis to treatment and follow-up [6]. Advances in biomarker discovery and validation continue to drive innovations in disease management, personalized medicine, and healthcare delivery, ultimately improving patient outcomes and quality of life. Genomic and proteomic data have revolutionized the field of biomarker discovery by providing comprehensive insights into the molecular mechanisms underlying diseases and identifying potential markers for diagnosis, prognosis, and therapeutic targeting [7]. A novel targeting approach is to recommend a multi-panel approach using the proteome of a patient, which represents the entire set of proteins expressed by a genome, tissue, or cell at a specific time under defined conditions [8]. Alterations in the proteome can reflect changes in cellular function, signaling pathways, and disease states. Understanding the proteomic landscape associated with various diseases has become crucial for identifying biomarkers, elucidating disease mechanisms, and developing targeted therapeutic interventions [8].

Biomarkers can be classified based on various criteria, including their origin, type, clinical utility, and application in disease diagnosis, prognosis, and therapeutic monitoring. Based on their application, we can identify three kinds of subclasses [9]. The first one comprises screening biomarkers, which are useful for screening programs to identify individuals at an increased risk of developing specific diseases. In this field, the most studied and characterized of these are the cancer screening biomarkers that are associated with tumor development; like other neoplasia, this is associated with inflammation [10], which is a normal and quick response to acute tissue damage resulting from physical injury, ischemic injury, toxins, or other types of injury [10]. The second one includes surrogate biomarkers, which substitute clinical endpoints, such as glycemia in type two diabetes diagnosis; the third subclass comprises all biological variation induced by a specific therapy [11,12]. Since most human disorders fall under a combination of genetic, epigenetic, and environmental factors, the identification of particular markers able to fully explain the complex “phenotype” of a disease in question is now the goal of the majority of research studies [13].

As is well known, a common trait of all human disorders is inflammation, since the various *interplayers* of human diseases trigger inflammatory signaling. The term inflammation is derived from the Latin word “inflammo”, which means “I set alight; I ignite”. As part of the body’s defense response, it is initially beneficial, but in chronic states characterized by low-grade chronic inflammation, it becomes detrimental and can be considered a causative factor that promotes the worst human disorders, including cancer, cardiovascular diseases, infection, and degenerative diseases [13]. Thanks to the advances in systems biology, both basic and clinical studies have attempted to map the initial phase of the inflammatory response pathway of various disorders, with the intent of reconstructing the molecular networks that characterize each disorder [14]. It is earnestly hoped that the studies collected in this Special Issue can establish a base for identifying new biological markers of human diseases, thus aiding in the ongoing efforts to better prevent, diagnose, and treat human disorders.
